# Association of *RAD51 *and *XRCC2* Gene Polymorphisms with Cervical Cancer Risk in the Bangladeshi Women

**DOI:** 10.31557/APJCP.2021.22.7.2099

**Published:** 2021-07

**Authors:** Sanjida Chowdhury Ivy, Samia Shabnaz, Mohammad Shahriar, Sarah Jafrin, Tutun Das Aka, Md. Abdul Aziz, Mohammad Safiqul Islam

**Affiliations:** 1 *Department of Pharmacy, University of Asia Pacific, Dhaka, Bangladesh. *; 2 *Department of Pharmacy, Noakhali Science and Technology University, Noakhali, Bangladesh. *

**Keywords:** Cervical cancer, RAD51, XRCC2, Polymorphism, Bangladesh

## Abstract

**Objective:**

Alterations in common DNA repair genes (*RAD51* and *XRCC2*) may lead to cervical cancer (CC) development. In the present study, we analyzed the association between *RAD51* rs1801320 and *XRCC2* rs3218536 polymorphisms and CC.

**Methods:**

Variants were selected based on their associations with some cancers in several ethnicities and the risk allele frequency (>0.05) in different populations. The variants were detected using the PCR-RFLP method. Adjusted odds ratios (aOR) and 95% confidence intervals (CI) were determined by logistic regression models.

**Result:**

Significantly increased risk (p<0.05) were detected for both SNPs with CC (rs1801320- GC vs. GG: aOR=2.21, 95% CI=1.43-3.42; CC vs. GG: aOR=4.48, 95% CI=1.76-11.42; dominant model: aOR=2.49, 95% CI=1.65-3.76; recessive model: aOR=3.52, 95% CI=1.40-8.88; allele model: OR=2.30, 95% CI=1.63-3.26, and rs3218536- GA vs. GG: aOR=2.77, 95% CI=1.85-4.17; AA vs. GG: aOR=5.86, 95% CI=2.08-16.50; dominant model: aOR=2.97, 95% CI=1.99-4.42; recessive model: aOR=3.56, 95% CI=1.30-9.73; and allele model: aOR=2.21, 95% CI=1.62-3.00). Besides, older patients (>60 years) with rs1801320 showed significantly reduced risk (OR=0.53, 95% CI=0.29-0.96, p=0.04) but with rs3218536 depicted significantly increased risk (aOR=2.44, 95% CI=1.20-4.96, p=0.01) for CC.

**Conclusion:**

This study indicates an association of rs1801320 and rs3218536 polymorphisms with CC and confirms that patients older than 60 years are more likely to develop CC for rs3218536 polymorphism.

## Introduction

Cervical cancer (CC), a major female malignant disease developed in the cervix of women’s uterus, is the overall seventh and fourth most frequently appeared female cancer worldwide based on its incidence and mortality rate (Arbyn et al., 2020; Hamadani et al., 2017). In 2018, nearly half a million CC patients (570,000 cases) were diagnosed with the death of 311,000 patients worldwide, while a larger number (approximately 80%) are from developing countries, including various Asian (southern, central and southeast regions), Caribbean and African countries with the highest mortality rate, mostly due to their delayed diagnosis (Hasan et al., 2021; Patil et al., 2018; Bray et al., 2018). Cervical cancer is the leading cause of cancer death among Bangladeshi women, and over 11,000 new CC cases have been identified every year, while more than 6,500 have died of this malignancy (Muhammad et al., 2021; World Health Organization, 2020; Ansink et al., 2008).

Clinical and epidemiological data demonstrate that various etiological factors can be responsible for this malignancy, including first intercourse at an early age, infection by different strains of sexually-transmitted Human Papillomavirus (HPV), alcohol consumption or smoking, various reproductive factors, sexual patterns, or multiple pregnancies, oral contraceptives, diets, excess weight, age, and so on (Das et al., 2021; Patil et al., 2018). Although the CC development is closely related to high-risk HPV infection, all HPV infected cases do not finally develop cervical cancer. Therefore, multiple molecular dysfunctions, along with the oncogenic activation or tumor suppressor gene inactivation, are also necessary for CC formation (Settheetham-Ishida et al., 2020; Chen et al., 2017).

Deoxyribonucleic acid (DNA), a core component of the cellular nucleus, usually suffers from numerous damaging agents- like radiations, sort of endogenous elements, and a number of chemicals, resulting in single-strand breaks (SSBs). Consequently, unrepaired SSBs bring about double-strand breaks (DSBs)- the most harmful form of damage, over the DNA replication stage of the cell cycle (Khanna and Jackson, 2001). After that, aggregation of these unrepaired DSBs can influence cell death with the initiation of malignancy that indicates an error in the DNA repair system, which plays a principal role in tumorigenesis. Homologous recombination repair (HRR)- one of the vital DSBs repairing mechanisms, is a key pathway that acts in the S phase of mammalian cell cycles (Lengauer et al., 1998). In previous studies, researchers reported that *RAD51* paralogs (*XRCC2*, XRCC3, *RAD51*B, *RAD51*C, and *RAD51*D) serve as a vital functioning protein during the process of HRR (Suwaki et al., 2011). Furthermore, it also revealed that any defect in HRR was found to be closely associated with multiple cancer formation in humans (Cerbinskaite et al., 2012). 

*RAD51*, a highly reserved gene appearing in maximum eukaryotes (from human to yeast), is homologous to the RacA and *RAD51* genes of E. coli and yeast, respectively. It encodes *RAD51* protein- a >39kb protein that holds 339 amino acids in chromosome 15q15.1, which is essential for DNA damage repair. It mainly involves the repair system of DSBs in DNA by HRR mechanisms (Korak et al., 2017; Nogueira et al., 2012; Kowalczykowski, 1991). This protein also directly interact with other genes- like *XRCC2*, *XRCC3, BRCA1, BRCA2*, to make a complex that is fundamental during the repairing and cross-linking of DNA, and in this manner, it plays a crucial role in the maintenance of chromosomal stability with cell cycle regulation (in both; meiotic and mitotic) and apoptosis (Tulbah et al., 2016; Paulíková et al., 2013). The rs1801320 (also called 135G>C, the substitution of G→C at 135th position) is a common single nucleotide polymorphism (SNP) of the *RAD51* gene that is situated in its 5’ untranslated region (5’UTR) (Nowacka-Zawisza et al., 2015). Previous studies recognized that the overexpression of the *RAD51* gene (rs1801320) could trigger the development of various cancers like breast and cervical cancer in humans (Zhang et al., 2012; Jara et al., 2007).

X-ray repair cross-complementing group 2 (*XRCC2*) gene is a highly conserved *RAD51* paralog that encodes an *XRCC2* protein, which forms a complex after getting together with another some proteins (like *RAD51*L3) and plays a pivotal role during chromosome segregation along with apoptotic response towards DSBs (Braybrooke et al., 2000; Griffin et al., 2000). Johnson et al. (1999) confirmed that this protein possesses a crucial relationship with the HRR process. Researchers found that SNPs in the DNA repair gene could modify the repair capacity of defected DNA and subsequently prompt cancer susceptibility, although the exact function remains unclear (Clarkson and Wood, 2005). The rs3218536 (G→A polymorphism) is a relatively rare SNP that is located in exon 3 of the *XRCC2* gene. After a nonsynonymous substitution in this variant, histidine (His) takes the place of arginine (Arg) at codon 188 that is also named as Arg188His (He et al., 2014a). This changed amino acid sequence influences the function of DNA repair systems that helps to develop cancer in different body parts (breast, ovary, and cervix) (Kamali et al., 2017). 

Here, we hypothesized that both *RAD51* rs1801320 and *XRCC2* rs3218536 are associated with risk of cervical cancer development and performed this case-control study of in the population of Bangladesh to identify the association with the development of cervical cancer.

## Materials and Methods

*Ethical approval*

This study was authorized by the ethical committee of the National Institute of Cancer Research and Hospital (NICRH), Mohakhali, Dhaka, and the University of Asia Pacific, Dhaka, Bangladesh. Before starting the investigation, written consent was received from all voluntarily recruited CC patients and healthy controls after notifying the purpose and all experimental study actions. 

*Study subjects*

From early to mid-2019, a total of 225 genetically unconnected ethnic Bangladeshi patients of all stages of uterine cervix carcinoma were gradually taken from the National Institute of Cancer Research and Hospital. Those histopathologically assured CC cases came from multiple regions of Bangladesh. During the presence of a gynecologist with two skilled nurses, all information (age, weight, height, and smoking status) were collected after entire diagnostic procedures with a proper personal interview. Besides, 199 healthy unrelated Bangladeshi women with negative cervical carcinoma were recruited from different parts of Bangladesh after matching of age with the patients. Some common inclusion criteria were followed for all the study subjects, including a) had confirmed with CC through diagnosis; b) no previous history of other cancers; c) genetically unrelated; d) age of more than 21 years. On the other hand, exclusion criteria followed are- a) age must not be under 21 years; b) unable to provide data; c) have concurrent chronic diseases; d) unwilling to participate e) patients were freshly diagnosed, and chemotherapy was not started before blood sample collection. Properties of all selected subjects (cases and controls) were recorded in a previously structured questionnaire form. This analysis was performed based on the Helsinki Declaration and its added amendments (World Medical Association Declaration of Helsinki, 2008). Overall experimental procedures were accomplished in Pharmacogenetics Laboratory, Department of Pharmacy, University of Asia Pacific (UAP), Dhaka, Bangladesh. 

*SNP selection*

From numerous identified polymorphisms of HRR pathway, the functionally potent polymorphisms (rs1801320 and rs3218536) of the investigation were sorted out from National Center for Biotechnology Information (NCBI) depending on some criteria, which are: (1) these SNPs were found to be associated with some carcinomas in several ethnicities and (2) the risk allele frequency (RAF) is >0.05 in different populations (Zerbino et al., 2018).

*DNA extraction and genotyping*

**Figure 1:**
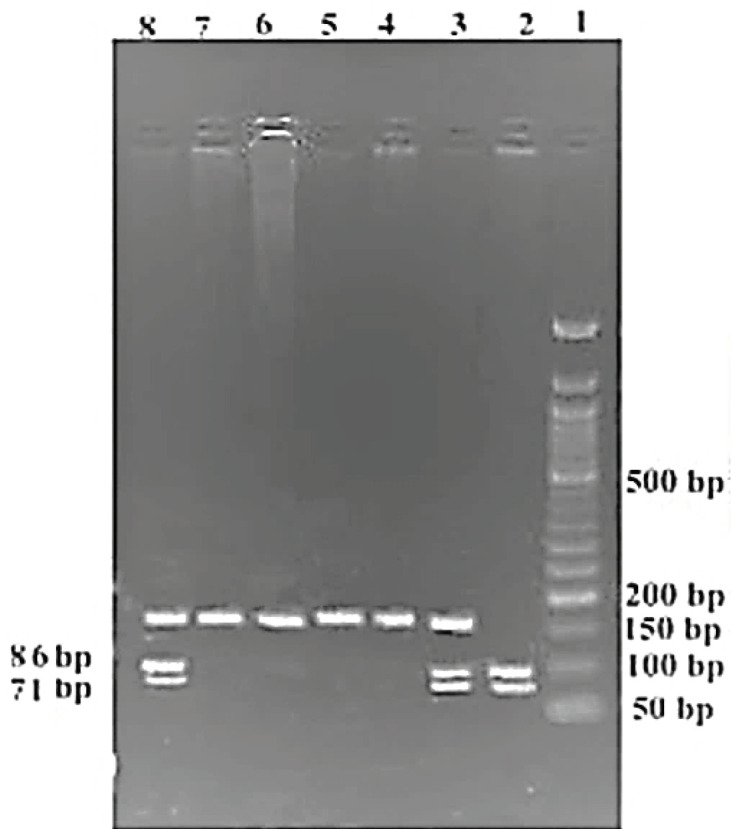
PCR-RFLP of *RAD51 *rs1801320 in 2% Agarose Gel; Lane-1 contains 50 bp ladder; lane 2 indicates GG genotype; lane 3, 8 indicate GC genotype; lane 4-7 indicate CC genotype

**Figure 2:**
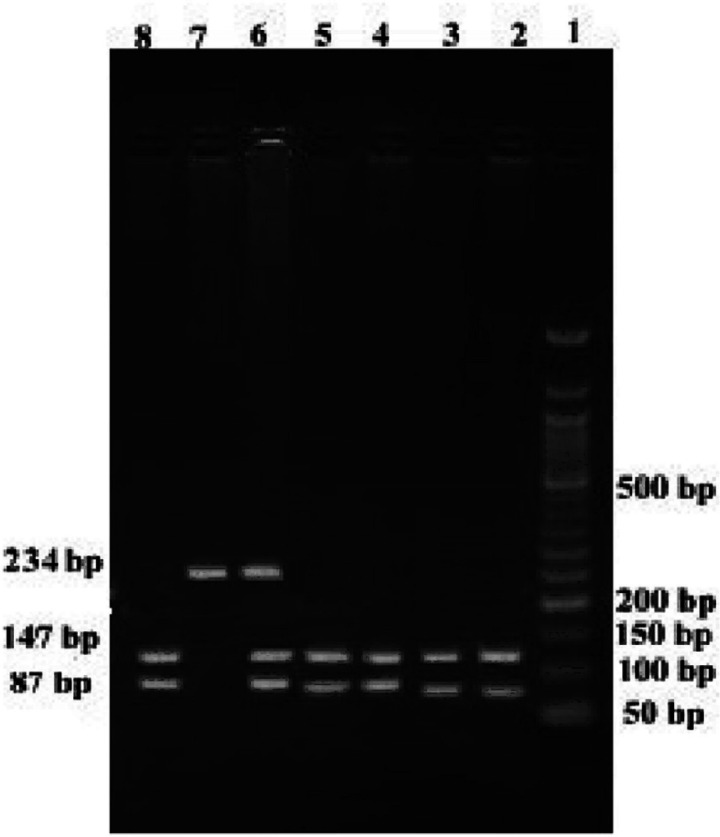
PCR-RFLP of *XRCC2* rs3218536 in 2% Agarose Gel; Lane-1 contains 50 bp ladder; lanes 2-5, 8 indicate AA genotype; lane 6 indicates GA genotype; lane 7 indicates GG genotype

From all recruited subjects, approximately 3 ml of peripheral blood were taken by venipuncture in ethylenediaminetetraacetic acid (EDTA)-Na2 containing vacutainer sterile tubes, and then genomic DNA was extracted by using FavorPrepTM Blood Genomic DNA Extraction Kit (Favorgen Biotech Corporation, Taiwan) according to their mentioned process. All extracted DNA were reserved at 40C temperature and then subjected to quantification. Both SNPs [rs1801320 (G>C) and rs3218536 (G>A)] were genotyped by the polymerase chain reaction-restriction fragment length polymorphism (PCR-RFLP) method. We have used predesigned primers for *RAD51* rs1801320 and *XRCC2* rs3218536, which can be found in a recently published article (Hridy et al., 2020) from our research group. All utilized primers, PCR conditions, expected PCR products, and digested fragments with restriction endonucleases are illustrated in Table 1. PCR products were confirmed after 1% agarose gel electrophoresis, and the digested PCR products were visualized on 2% agarose gel after dyeing with ethidium bromide (Figure 1, Figure 2). To confirm the results, a subset of heterozygous and homozygous mutant samples (20%) was tested twice, and 100% concordance was observed in our findings.

*Statistical analysis*

The odds ratios (ORs) and 95% confidence intervals (95% CI) for all genotypes were evaluated by MedCalc software (version 12.1.4) to get the risk of association of both variants. We adjusted ORs and 95% CI with age. Adjusted odds ratios (aOR) and 95% CI were measured by logistic regression models. Besides, any dislodgment from Hardy–Weinberg equilibrium (HWE), and all other statistical analysis was conducted using SPSS (version 20), and p <0.05 was considered statistically significant. Statistical power was measured using the online sample size estimator (OSSE) tool for each SNP (http://osse.bii.a-star.edu.sg/).

## Results

*Clinical distributions of cases and controls*

A total of 225 female cervical cancer cases and 199 healthy female volunteers were recruited for this case-control study. The mean age of the cases and controls was 57.64 and 52.54 years consecutively. Among the CC patients and controls, 65.33% and 50.5% were between the ages of 45 and 60 years, while 27.56% and 41.71% were higher than 60 years old, respectively. The distribution of clinical variables among all selected study participants is mentioned in Table 2. Additionally, 8.44% were overweight patients from all the cases, and their average BMI was 26.53 ± 0.85, while 91.56% were normal patients, and the average BMI was 19.02 ± 2.89. None of the selected subjects was involved in smoking. From the history of contraception, it was found that 55.11% of patients and 45.23% of controls regularly took oral contraceptive pills (OCPs).

*Association of rs1801320 and rs3218536 with cervical cancer*

The association between rs1801320 and rs3218536 polymorphisms with the development of CC was presented in Table 3. After the adjustment with ages, there were no mentionable differences between the adjusted odds ratio (aOR) and crude odds ratio as well as in p-values. In case of rs1801320, GC carriers were significantly associated with the formation of CC (GC vs. GG: aOR = 2.21, 95% CI = 1.43-3.42, p = 0.0004), while in dominant model, GC+CC carriers were also connected an elevated chance of CC formation (GC+CC vs GG: aOR = 2.49, 95% CI = 1.65-3.76, p <0.0001). Equivalently, CC carriers were related with a highly increased risk of cervical cancer development (CC vs. GG: aOR = 4.48, 95% CI = 1.76-11.42, p = 0.002). Likewise, we got a statistically notable correlation with a higher risk of CC formation in case of recessive model (CC vs. CG+GG: aOR = 3.52, 95% CI = 1.40-8.88, p = 0.008) and C allele (C vs. G: OR = 2.30, 95% CI = 1.63-3.26, p <0.0001). Additionally, the patients and controls with this SNP showed no deviation from HWE (p = 0.179 and 0.312, respectively).

Furthermore, in case of rs3218536, it is distinctly visible (Table 3) that GA carriers significantly correlated with increased chances of CC progression (GA vs. GG: aOR = 2.77, 95% CI = 1.85-4.17, p <0.0001) and in the same manner, GA+AA and AA carriers also illustrated a significant association with CC in dominant (GA+AA vs. GG: aOR = 2.97, 95% CI = 1.99-4.42, p <0.0001) and recessive model (AA vs. GA+GG: aOR = 3.56, 95% CI = 1.30-9.73, p = 0.013). Similarly, we got a statistically remarkable association with a greatly elevated risk of CC formation in case of AA genotype (AA vs. GG: aOR = 5.86, 95% CI = 2.08-16.50, p = 0.001) and A allele carriers (A vs. G: OR = 2.21, 95% CI = 1.62-3.00, p <0.0001). Besides, although healthy controls of this SNP displayed consistency with HWE (p = 0.096) but study patients showed deviation over here (p = 0.001).

*Association of rs1801320 and rs3218536 with overweight CC cases*

In order to find out the relationship between excess weight and CC development, we evaluated the association of both SNPs among overweight and normal weight CC patients as shown in Table 4. In the case of rs1801320, GC, CC, and GC+CC (from dominant model; GC+CC vs. GG) carriers did not show any significant association, but presented higher risk of CC formation (OR = 2.21, 1.61, 2.08; 95% CI = 0.81-6.07, 0.31- 8.34, 0.79-5.50 and p = 0.12, 0.57, 0.14, respectively). Likewise, in case of rs3218536, GA carrier found no notable association with no risk in CC development (OR = 0.99, 95% CI = 0.35-2.89 and p = 0.99), whereas AA and GA+AA (from dominant model; GA+AA vs. GG) carrier also didn’t find statistically significant association, but got the higher chances of CC formation (OR = 2.22, 1.14; 95% CI = 0.50-9.83, 0.42-3.13; and p = 0.29, 0.80, gradually). 

*Correlation of both SNPs with the age of selected cases*

Different age groups were compared with the data of different allele carriers including combined effect of heterozygote and mutant homozygote (HE+MH) and normal homozygote (NH) of studied cases (Table 5). After the evaluation, in case of rs1801320 and rs3218536, we found lower chances of CC formation in the age group of <45 (OR = 0.67, 1.16; 95% CI = 0.23-1.90, 0.39-3.46; and p = 0.45, 0.80, by turn) and 45-60 (OR = 1.29, 0.74; 95% CI = 0.85-1.97, 0.48-1.14; and p = 0.24, 0.18, gradually) years but the results were statistically not significant. Besides, in the group of >60 years CC cases, a lower but significant risk obtained in case of rs1801320 (OR= 0.53, 95% CI= 0.29-0.96 and p = 0.04), while statistically significant association with higher chance of CC development found for rs3218536 (OR= 2.44, 95% CI = 1.20-4.96 and p = 0.01).

*Statistical power calculation*

For both the SNPs, statistical power was determined using the minor allele frequency (MAF) of the cases and controls with the OSSE online sample size estimator at a 5% significance level. For the SNPs rs1801320 and rs3218536, the power obtained was 90.5% and 95.4%, respectively.

**Table 1:** Primers, PCR Conditions and Restriction Enzymes with Digestion Condition of the Selected Alleles of *RAD51* and *XRCC2* genes

SNPs	Primers	GC (%)	PCR condition	No. of cycles	SAF (bp)	RE	Digestion condition	Expected fragment
*RAD51 rs1801320*	FP: 5’-TGGGAACTGCAACTCATCTGG-3’	52.4	95°C 30s	35	157	BstNI	60°C (incubated for 4 hours)	NH:GG: 71,86
			66.8°C 30s					HE:GC :71,86,157
	RP: 5’-GCGCTCCTCTCTCCAGCAG-3’	68.4	72°C 60s					MH:CC: 157
*XRCC2 rs3218536*	FP: 5'- TTGCTGCCATGCCTTACAGA -3'	50	95°C 30s	35	234	Hph1	37ºC (incubated overnight)	NH:GG: 234
			51.9°C 30s					HE:GA: 87,147,234
	RP: 5'- TGGATAGACCGCGTCAATGG -3'	55	72°C 60s					MH: AA: 87,147

SNPs, single nucleotide polymorphisms; GC, guanine-cytosine; FP, Forward primer; RP, Reverse primer; SAF, size of amplification fragment; BP, base pair; RE, restriction endonuclease; NH, normal homozygote; H, heterozygote; MH, mutant homozygote

**Table 2:** Clinical Characteristics of Cervical Cancer Cases and Controls

Variables	Cases, n (%)	Controls, n (%)
Number	225	199
Age (Years)		
Mean age	57.64	52.54
<45	16 (7.11)	15 (7.4)
45-60	147 (65.33)	101 (50.75)
>60	62 (27.56)	83 (41.71)
BMI (kg/m^2^) (±SD)		
Overweight		N/A
	19 (8.44)	
	26.53 ± 0.85	
Normal		N/A
	206 (91.56)	
	19.02 ± 2.89	
Smoking status	No	No
Contraception		
Oral pills	124 (55.11)	90 (45.23)
Others	20 (8.89)	40 (20.10)
None	81 (36)	69 (34.67)

BMI, body mass index; NA, not available

**Table 3:** Association of Several Genotypes and Alleles of rs1801320 and rs3218536 with Cervical Cancer

Polymorphisms	Genotype/Allele	Cases (n=225) (%)	Controls (n=199) (%)	OR (95% CI)	p-value	aOR (95% CI)	p-value
*RAD51 rs1801320*	GG	120 (53.33)	147 (73.87)	1	-	1	-
	GC vs. GG (Codominant 1)	83 (36.89)	46 (23.12)	2.21 (1.43-3.41)	**0.0003**	2.21 (1.43-3.42)	**0.0004**
	CC vs. GG (Codominant 2)	22 (9.78)	6 (3.02)	4.49 (1.76- 11.43)	**0.0016**	4.48 (1.76-11.42)	**0.002**
	Dominant model (GC+CC vs. GG)	105 (46.67)	52 (26.13)	2.47 (1.64-3.73)	**<0.0001 **	2.49 (1.65-3.76)	**<0.0001**
	Recessive model (CC vs. CG+GG)	22 (9.78)	6 (3.01)	3.49 (1.38 - 8.78)	**0.0081**	3.52 (1.40-8.88)	**0.008**
	C allele	127 (28.22)	58 (14.57)	2.30 (1.63-3.26)	**<0.0001**	-	**-**
*XRCC2 rs3218536*	GG	77 (34.22)	120 (60.30)	1	**-**	1	**-**
	GA vs. GG (Codominant 1)	129 (57.33)	74 (37.19)	2.72 (1.81-4.07)	**<0.0001**	2.77 (1.85-4.17)	**<0.0001**
	AA vs. GG (Codominant 2)	19 (8.44)	5 (2.51)	5.92 (2.12-16.52)	**0.0007**	5.86 (2.08-16.50)	**0.001**
	Dominant model (GA+AA vs. GG)	148 (65.78)	79 (39.70)	2.91 (1.97-4.34)	**<0.0001**	2.97 (1.99-4.42)	**<0.0001**
	Recessive model (AA vs. GA+GG)	19 (8.44)	5 (2.51)	3.58 (1.31-9.77)	**0.013**	3.56 (1.30-9.73)	**0.013**
	A allele	167 (37.11)	84 (21.11)	2.21 (1.62-3.00)	**<0.0001**	-	**-**

OR, Odds ratio: aOR, Adjusted odds ratio; 95% Cl, 95% confidence interval; p <0.05 is significant (bold)

**Table 4:** Correlation of rs1801320 and rs3218536 Polymorphisms with Overweight Cervical Cancer Cases

Polymorphisms	Risk allele	RAF	Genotype	Cases with overweight (n=19) (%)	Cases with normal weight (n=206) (%)	OR (95% CI)	p-value
*RAD51* rs1801320	C	0.37	GG	7 (36.84)	113 (54.85)	1	-
			GC	10 (52.63)	73 (35.44)	2.21 (0.81-6.07)	0.12
			CC	2 (10.53)	20 (9.71)	1.61 (0.31- 8.34)	0.57
			GC+CC	12 (63.16)	93 (45.15)	2.08 (0.79-5.50)	0.14
*XRCC2* rs3218536	A	0.42	GG	6 (31.58)	71 (34.47)	1	-
			GA	10 (52.63)	119 (57.76)	0.99 (0.35-2.89)	0.99
			AA	3 (15.79)	16 (7.77)	2.22 (0.50-9.83)	0.29
			GA+AA	13 (68.42)	135 (65.53)	1.14 (0.42-3.13)	0.8

RAF, risk allele frequencies; OR, odds ratio; 95% Cl, 95% confidence interval; p <0.05 is significant

**Table 5:** Correlation of rs1801320 and rs3218536 Polymorphisms with Older Cervical Cancer Cases

Characteristics	Cases	*RAD51* rs1801320				*XRCC2* rs3218536			
		HE+MH	NH	OR (95 % CI)	p-value	HE+MH	NH	OR (95 % CI)	p-value
Age (Years)									
<45	16	6	10	0.67 (0.23-1.90)	0.45	11	5	1.16 (0.39-3.46)	0.8
45-60	147	79	68	1.29 (0.85-1.97)	0.24	86	61	0.74 (0.48-1.14)	0.18
>60	62	20	42	0.53 (0.29-0.96)	0.04	51	11	2.44 (1.20-4.96)	0.01
45-60 + >60	209	99	110	Ref.	-	137	72	Ref.	-

HE, heterozygous; MH, mutant homozygous; NH, normal homozygous; OR, odds ratio; 95% Cl, 95% confidence interval; p <0.05 is significant; Bold values are identified as statistically significant.

## Discussion

Despite the drastically increased incidence of cervical cancer, the precise reason for its development is unrevealed until today. It is presumed that, like in the case of all other cancers, the growth of cervical cancer is a consequence of reciprocal action between the genetic predisposition and environmental factors. After analyzing the last few decades, researchers found that DNA repair pathways are continuously monitoring and correcting the exogenous and endogenous mutagens created damages in chromosomes. By repairing those defects, these pathways play an important role in protecting from genetic alterations (Datta et al., 2020; Bernstein et al., 2013; Dixon et al., 2004). HRR is one of those pathways, and extensive genetic studies of this pathway have been conducted to identify susceptible loci of different cancer types (Tulbah et al., 2016). This retrospective study demonstrates an explicit confirmation of *RAD51* rs1801320 and *XRCC2* rs3218536 polymorphisms association with CC development in Bangladeshi women.

The repair of breakage in DNA double-strand is properly accomplished by homologous recombination. This action is promoted through recombinase *RAD51* that catalyze the premier reactions of HRR and protect DNA strands from severe invasion. Various members of the RAD family (including previously mentioned paralogs) co-operate the *RAD51* gene in this process, and all these genes are highly polymorphic (Walsh, 2015; Krejci et al., 2012; San Filippo et al., 2008). The rs1801320 (135G>C, also named c. -98G>C) is one of the very common polymorphisms in *RAD51* that is located at 5´UTR. Even though the functional impacts of c. -98G>C polymorphism is unclear, but it is hypothesized that it can control the *RAD51* expression with the level of mRNA in as much as it alternates the pattern of CpG island in the promoter region (Antoniou et al., 2007; Hasselbach et al., 2005). It was obtained that the rs1801320 SNP in *RAD51* is correlated with an increased risk of ovarian (Smolarz et al., 2013; Romanowicz-Makowska et al., 2012), breast (Sun et al., 2011), colorectal (Nissar et al., 2014), endometrial (Michalska et al., 2014), head and neck (Sliwinski et al., 2010), myelodysplastic syndrome (He et al., 2014), Fuchs and keratoconus endothelial corneal dystrophy (Synowiec et al., 2013), as well as triple-negative breast cancer (Smolarz et al., 2013) in several ethnicities. No association study between *RAD51* polymorphisms and cervical malignancy has been reported yet except Zhang et al., (2012), where they found *RAD51* (135G>C) as a potential risk factor for cervical intraepithelial neoplasia development in the Chinese population (OR = 4.246; p = 0.014). 

The *XRCC2* gene is recognized as a vital portion of HRR pathway. It is needed for proper segregation of chromosomes and apoptotic reaction to DSB (Nowacka-Zawisza et al., 2015). This gene was cloned by mutant cell lines complementation of hamster that is hypersensitive to various DNA-damaging agents like ionizing radiation. Subsequently, *XRCC2* protein has been demonstrated to be essential for HRR and is needed for the formation of *RAD51* focus (Rafii et al., 2002). Different polymorphisms of this gene cause embryonic lethality, and it has a more micro effect on the repair capacity of DNA. Significant numbers of SNPs were detected in the gene *XRCC2*, where rs3218536 (c.563G > A; Arg188His) is an important and most commonly studied SNP for various forms of cancer (Pérez et al., 2013). It was observed that a large number of analyzes have attempted to detect the association between rs3218536 polymorphism of *XRCC2* gene and different human cancers particularly, ovarian (Auranen et al., 2005), colorectal (Curtin et al., 2009), thyroid (García-Quispes et al., 2011), endometrial (Han et al., 2004a), pancreatic (Jiao et al., 2007), lung (Zienolddiny et al., 2006), upper aerodigestive tract (UADT) (Benhamou et al., 2004), brain (He et al., 2014b), and skin (Han et al., 2004b) cancer in different populations. However, a notable association was not found in the case of all cancer types. Besides, Pérez et al., (2013) found *XRCC2* rs3218536 as a potent risk factor for cervical cancer development in Argentine women (OR = 2.4; p = 0.02), though Datkhile et al., (2018) did not find any prominent association in the rural population of Maharashtra, India (OR = 0.94; p = 0.77). 

Overweight, along with obesity, a multifactorial and largely preventable complex disease. By 2030, if present trends continue, approximately 38% of the adult population all over the world will be overweight. Many researchers stated that excess weight is associated with malignancy, and in 2007, almost 6% of all diagnosed cancers (men: 4%; women: 7%) were attributed to overweight within obesity. Besides being a major predisposing factor for diabetes, it also acts as a risk factor in the case of most cancer, including colon, postmenopausal breast, renal, endometrial, pancreatic, and esophageal cancers. Before some ages, evidence has deposited that overweight raises the risk of cancer formation in the liver, gallbladder, ovaries, blood, as well as prostate gland (Hruby and Hu, 2015). Furthermore, some analysis has reported a correlation of overweight with elevated incidence and mortality of CC. Additionally, in American women (specifically from Northern California), Clarke et al., (2018) observed a higher chance of CC formation with increasing BMI, and they also mentioned that excess BMI is related to reduced efficacy of cervix melanoma screening though its mechanism is unknown. 

Malignancy in the cervix is generally a disease of older and middle-aged women. Numerous studies showed that older females are suffered from advanced-stage disease during the diagnosis. Ashley suggested before that the age-associated incidence pattern of CC assembles to two components, where one form occurs at young ages that grow slowly and positively responds to treatment; another one appears at older ages and shows a poor response to treatment (Ashley, 1966a; Ashley, 1966b). In Japan, older women were found with more advanced CC and had a silly treatment outcome. In Europe, the number of CC diagnosed elderly patients regularly increases, while >40% of older women died from CC annually (Gao et al., 2013). 

The outcome of the current study demonstrates that *RAD51* rs1801320 and *XRCC2* rs3218536 polymorphisms significantly associated with 4-times and 5-times higher chances, respectively, of CC development in the Bangladeshi population. Statistically significant correlation in all mutant allele carriers (dominant model) were identified between CC and control group (p <0.0001 in both SNPs). No prominent association was found between overweight and CC for both polymorphisms (135G>C and 563G>A), but it showed a higher risk of cancer formation (OR = 1.61 and 2.22, by turn). Moreover, both SNPs showed a significant correlation in the case of older patients (>60 years). However, rs1801320 polymorphism showed the protective effect to CC; the other polymorphism, rs3218536, displayed a statistically notable association with a greater chance of cancer formation in the cervix of older women (OR = 2.44, p = 0.01).

However, our study has some limitations that should be addressed. Firstly, during the selection of SNPs, we have chosen only recognized SNPs from a public database. Secondly, the number of samples studied in this investigation is somehow lower than other published studies, which may influence the statistical significance to some extent. Thirdly, due to the unavailability of proper clinical information, we could not include some key data, including HPV infection or the influence of environmental factors. Therefore, future research with large sample size is recommended to validate this result.

In conclusion, this study indicates an association of *RAD51* rs1801320 and *XRCC2* rs3218536 polymorphisms with cervical cancer development in the Bangladeshi population. Moreover, our study confirmed that patients older than 60 years are more likely to develop cervical cancer. Further work with a broad population and gene-gene and the interaction between gene and environment are required to confirm these findings.

## Author Contribution Statement

S.C. Ivy: Performed the experiments; Analyzed and interpreted the data; wrote the paper. M. Shahriar and S. Shabnaz: Analyzed and interpreted the data; wrote the paper. T.D. Aka and S. Jafrin: Performed the experiments; Contributed re-agents, materials, analysis tools, or data; wrote the paper. M.A. Aziz: Analyzed and interpreted the data; Contributed reagents, materials, analysis tools or data; Wrote the paper. M.S. Islam: Conceived and designed the experiments; Analyzed and interpreted the data; Contributed reagents, materials, analysis tools or data; Wrote the paper.

## Study approval

Ethical committee of the University of Asia Pacific, Dhaka, Bangladesh approved the study protocol as a part of thesis work of S.C. Ivy.
